# Weighted multiple testing procedures for genomic studies

**DOI:** 10.1186/1756-0381-5-4

**Published:** 2012-06-07

**Authors:** Jiang Gui, Tor D Tosteson, Mark Borsuk

**Affiliations:** 1 Section of Biostatistics and Epidemiology, Department of Community and Family Medicine, Geisel School of Medicine, Lebanon, NH, USA; 2 Thayer School of Engineering, Dartmouth College, Hanover, NH, USA; 3 Dartmouth-Hitchcock Medical Center, One Medical Center Dr., 883 Rubin Bldg, HB7927, Lebanon, NH 03756, USA

**Keywords:** False discovery rate, Family-wise error rate, Genomic studies

## Abstract

With the rapid development of biological technology, measurement of thousands of genes or SNPs can be carried out simultaneously. Improved procedures for multiple hypothesis testing when the number of tests is very large are critical for interpreting genomic data. In this paper, we review recent developments on three distinct but closely related methods involving p-value weighting to improve statistical power while also controlling for the false discovery rate or the family wise error rate.

## Introduction

In genome-wide studies and gene expression data analysis, thousands of hypothesis tests are carried out at the same time. To control type I error arising from multiple testing, Bonferroni correction [1] is used to determine the statistical significance level of individual hypotheses to ensure that the probability of any single false positive among all tests (the family wise error rate, FWER) is controlled at the nominal significance level. This strict criterion has been used primarily in studies where only a few null hypotheses are expected to be false.

In the context of high-dimensional data analysis, using a procedure that guards against any single false positive occurring can lead to additional missed findings i.e. increased Type II error rates. Benjamini and Hochberg [2] proposed a procedure to control the “false discovery rate” (FDR), which is defined as the proportion of null hypotheses that are rejected erroneously. This criterion is less stringent than equivalent FWER-based procedures and provides a useful compromise between the loss of power attributable to the Bonferroni correction and the lack of control of Type I errors associated with comparisons unadjusted for multiplicity. Much additional research has been done on this approach, including the proposal for the q-value method by Storey [3,4] as a generalization of the p-value to the FDR setting, and the local FDR introduced by Efron et al. [5-7]. The FDR method has been widely applied to microarray analysis to detect differentially expressed genes, and is incorporated into popular software packages, e.g. SAM (Significance Analysis of Microarrays) and LIMMA (Linear Models for Microarray Data) in R.

Although it improves Type II error rates relative to FWER-based methods, the FDR method still results in relatively low power when the number of tests is in the thousands. To further improve power, Genovese et al. [8] proposed extending the FDR method to include weighted p-values and proved that as long as the sum of weights equals the total number of tests, the weighted method still controls FDR at the nominal level. Further work has focused on alternative methods for selecting weights, including a data splitting technique proposed by Roeder and Wasserman [9]. In genomic studies, researchers have access to expert biological knowledge through public databases such as GO and KEGG. It would be advantageous to take this information into consideration to improve power. For this reason, methods for p-value weighting have become an active research area.

In this review, we first provide background on the concept of FDR, including the BH algorithm for FDR control and extensions employing p-value weighting to improve average power. We then review approaches for assigning optimal weights in several problem settings, including FWER and FDR control, as well as grouped FDR. We also describe example applications in which the techniques have been used for genomic studies. Finally we summarize these approaches, provide recommendations, and discuss future research directions.

## Background

Holm [10] proposed a generalized sequential multiple testing procedure to control FWER that introduced the p-value weighting idea. More recently, Roeder and Wasserman [9] demonstrated a weighted Bonferroni procedure to incorporate weighted p-values. In the same manuscript they derived the form of optimal weights that maximize the average power in terms of the unknown means when test statistics are normally distributed. The weighted p-value method was applied in the context of FDR control by Genovese et al. [8]. After introducing some notation, each of these is reviewed below, followed by a review of methods for determining optimal p-value weights.

### Notation

Let ***T*** = ( ***T***_**1**_, ***T***_**2**_, ⋯, ***T***_*m*_) denote test statistics for *m* hypotheses. The p-values associated with the tests are ***P*** = ( ***P***_**1**_, ***P***_**2**_, ⋯, ***P***_***m***_). Suppose that for ***m***_1_ tests the null hypothesis is true and for ***m***_2_ tests the alternative hypothesis is true. Let ***H***_**0**_ denote the set of all true null hypotheses and ***H***_**1**_ denote the set of all true alternative hypotheses. Table 1 defines the notation for variables summarizing the possible results for the hypothesis tests. Based on this notation, FWER and FDR are defined as: FWER = Pr(V≥1) and FDR = E(V/R) Pr(R>0).

**Table 1:** Possible results of tests of multiple hypothesis

	**# declared non-significant**	**# declared significant**	**Total**
**# true null hypotheses**	*U*	*V*	***m***_**1**_
**# true alternative hypotheses**	*Z*	*S*	***m***_**2**_
Total	*m* − *R*	*R*	*m*

### Benjamini and Hochberg’s FDR control procedure

Let ***P***_(**1**)_ <***P***_(**2**)_ < ⋯ <*P*_(*m*)_ be the ordered p-values from ***P***. Denote the hypothesis that corresponds to ***P***_(***i***)_ to be ***H***_(***i***)_. The Benjamini and Hochberg’s procedure (BH procedure) finds the largest ***iI***, satisfying ***P***_(***i***)_ <***iα***/ ***m*** and rejects all hypotheses that ***P*** <***P***_(***I***)_. This procedure controls FDR at level ***α*** ( **0** <*α* < 1) under independence or “positive regression dependency” for the test statistics corresponding to the true null hypotheses [2,11]. An example of positive regression dependency is when the test statistics all have positive pair wise correlations.

### Weighted BH procedure

Genovese et al. [8] proposed a simple weighted BH procedure (wBH) in which weights ***W***_***i***_ are assigned to each null hypothesis such that $\;\sum_{i=1}^{m}W_{i}=m$*.* The BH procedure is applied directly to ***Q***_***i***_ = ***P***_***i***_/***W***_***i***_. Arguments similar to those used for unweighted FDR show that wBH controls FDR at the nominal level.

### Weighted Bonferroni procedure

For control of the FWER, Roeder and Wasserman [9] proposed a weighted Bonferroni procedure in which weights ***W***_*i*_ are assigned to each null hypothesis such that $\;\sum_{i=1}^{m}W_{i}=m$. The Bonferroni procedure is then applied directly to ***Q***_***i***_ = ***P***_***i***_/***W***_***i***_

## Review of methods for optimal weighting

### Definition of average power

To develop optimal weighting strategies, it is useful to generalize the concept of power to the multiple testing setting by considering the average power of the*m*_2_ tests in which the alternative hypothesis is true. Assume that *T*_*j*_ ∼ *N*( *ξ*_*j*_, 1).If *H*_*j*_ is a false null hypothesis for a one-sided test, then *ξ*_*j*_ > 0. For simplicity, following the presentation in Roeder and Wasserman [9], only one-sided tests are considered in our review, although similar developments apply for two-sided tests. Let Φ(x) denote the standard normal cumulative distribution function. The power for a single test can be expressed as

(1) $$\pi \left(\xi_{j},{\text{ W}}_{\text{j}}\right)=\text{Pr}\left({\text{P}}_{\text{j}}<\frac{\alpha {\text{W}}_{\text{j}}}{\text{m}}\right)=\mathrm{Pr}\left({\text{T}}_{\text{j}}>\Phi^{-1}\left(\frac{\alpha {\text{W}}_{\text{j}}}{\text{m}}\right)\right)\;$$

Equation (1) can be further simplified as

(2) $$\pi \left(\xi_{j}\text{,}\;W_{j}\right)=\Phi \left(\Phi^{-1}\left(\frac{\alpha W_{j}}{m}\right)-\xi_{j}\right)\;$$

The average power is then defined as

(3) $$E\left(S/m\right)=\frac{1}{m}\sum_{j=1}^{m}\left(\xi_{j}\text{,}\;W_{j}\right)I\left(\xi_{j}>0\right)\;$$

In the following sections, we review methods for finding weights that maximize average power in three relevant problem settings, first for Bonferroni control of FWER, then for FDR, and finally for grouped FDR.

### Problem setting I: FWER control

Using the weighted Bonferroni procedure to control the FWER at level α, what is the *W* = ( *W*_1_, *W*_2_, ⋯, *W*_*m*_) that will maximize the average power?

Roeder and Wasserman [9] showed that the optimal “oracle” weight can be obtained by setting the derivatives of (3) to zero and solving the equations subject to W_j_ > 0 and $\;\sum_{i=1}^{m}W_{i}=m$. This leads to the following solution in terms of the unknown test means *ξ*_*i*_:

(4) $${\hat{W}}_{j}=\left(\frac{m}{\alpha }\right)\Phi \left(\frac{\xi_{j}}{2}+\frac{c}{\xi_{j}}\right)I\left(\xi_{j}>0\right)$$

where*c* is a constant so that

(5) $$\sum_{j=1}^{m}{\hat{W}}_{j}=m$$

Although the *ξ*_*j*_ are unknown, available data can be used to generate preliminary estimates. In the absence of data, it has been proposed to use a data-splitting approach [12] to provide such an estimate. If the data are identically and independently distributed then one can randomly split the data in two parts and use the first part as a training set to estimate *ξ*_*j*_ and the corresponding optimal weights. These are then applied to the testing set.

In a follow-up paper, Roeder et al. [13] applied data splitting weights in a genome association study. They pointed out that the power gain from the weighted procedure cannot compensate for the power loss resulting from the splitting the data and using only a fraction of all samples as the test set. Instead, they propose to form *k* groups of tests with sizes of perhaps 10–20 that are likely to have the same mean test statistics. Assuming that this procedure is only approximately well informed; the distribution of the test statistics in each group can be assumed to follow a normal mixture distribution based on the proportion of true and false null hypotheses. They suggest moment estimators for the common group test statistic non-zero means, ${\hat{\xi }}_{k}$, and the proportion of true null hypotheses, *π*_*k*_, and use these to develop the weights in using Equations (4) and (5). If ${\hat{\pi }}_{k}\leq \frac{1}{r_{k}}$, where *r*_*k*_ denotes the number of tests in the kth group, then ${\hat{\xi }}_{k}=0$. A smoothing procedure is proposed to account for excessive variability. They are able to show that this procedure controls FWER at level α*.* Software to implement this procedure can be found at http://wpicr.wpic.pitt.edu/WPICCompGen/.

To further demonstrate the merit of the proposed procedure, Roeder and colleagues showed in a simulation study that the grouped weighting procedure gains power when multiple tests with signals are clustered together in one or more groups. When the grouping is poorly chosen and many groups contain no true signal, the weights may not improve power, although in practice little power is lost under such circumstances.

### Problem setting II: FDR control

Using theweighted wBH procedure and controlling FDR at level α, what is the *W* that will maximize the average power?

Identifying optimal weights under FDR control is more difficult than in the FWER setting because FDR has a random variable (the number of rejections) in the denominator. Roquain and van de Wiel [14] proposed an indirect approach to tackle this problem. They first fix the rejection region then perform the optimization for each fixed rejection (Δ_*j*_ := *j* tests have been rejected) which in turn leads to a family of optimal weight vectors ( *W*_*i*_(*j*), *i* = 1, …, *m*).

Roquain and van de Wiel [14] give the following multi-weighted algorithm:

Step 1: Compute for each *i* the weight vector *W*_*i*_(*m*). If all p-values *P*_*i*_ are less than or equal to *αW*_*i*_(*m*), then reject all hypotheses. Otherwise go to step 2.

Step *j* ( *j* ≥ 2): Set *r* = *m* − *j* + 1 and compute for each *i* the weight vector *W*_*i*_(*r*) and the weighted p-value$\;P_{i}^{\prime }=\frac{P_{i}}{W_{i}\left(r\right)}$. Order the weighted p-values following $P_{\left(1\right)}^{\prime }\leq \cdots \leq P_{\left(m\right)}^{\prime }$. If $P_{\left(r\right)}^{\prime }\leq \mathrm{\alpha r}/m$, then reject the null hypotheses corresponding to the smaller weighted p-values $P_{1}^{\prime },1\leq i\leq r$. Otherwise go to step *j* + 1. When *j* = *m*, stop and reject none of the null hypotheses.

Note that if we set all weights to be 1, this procedure is reduced to the standard BH procedure. With the involvement of a single weight vector, this procedure can be reduced to the wBH procedure. The unique feature of multi-weighted linear step-up procedure is that it introduces several weight vectors corresponding to different rejection regions. This yields more flexibility than wBH procedure in term of boosting power. However, since this algorithm involves multiple weight vectors under different rejection regions, it cannot rigorously control FDR for any pre-determined weight matrix *W*. Therefore the following adjustment was suggested to control FDR.

Let ${\tilde{W}}_{i}\left(r\right)=\frac{W_{i}\left(r\right)}{1+\alpha W_{i}\left(m\right)}$ and replace *W*_*i*_(*r*) with $\tilde{W_{i}}\left(r\right)$in the above step-up procedure to control FDR at level α under the assumption that p-values are independent. Since *W*_*i*_(*m*) ≤ *m* and α is usually small, Roquain and van de Wiel argue that *W*_*i*_(*r*) and $\tilde{W_{i}}\left(r\right)$are close to each other and the small corrections can be ignored.

Under this multi-weighting framework, one can freely choose weight for any given rejection region. Since the FDR procedure’s cutoff with *r* rejections is *αr*/ *m*, the power can be defined similarly to (2), (3), simply replacing *α*/ *m* with *αr*/ *m*. The same logic follows for Equations (4). Therefore, the optimal weight for fixed rejection region *r* is:

(6) $${\tilde{\text{W}}}_{\text{j}}\left(\text{r}\right)=\left(\frac{\text{m}}{\alpha \text{r}}\right)\Phi \left(\frac{\xi_{\text{j}}}{2}+\frac{\text{c}(\text{r})}{\xi_{\text{j}}}\right)\text{I}\left(\xi_{\text{j}}>0\right)$$

where *c*( *r*)is a constant that satisfies:

(7) $$\sum_{\text{j}=1}^{\text{m}}\hat{{\text{W}}_{\text{j}}}\left(\text{r}\right)=\text{m}$$

Roquain and van de Wiel’s idea of fixing the rejection region and offering an algorithm to control FDR at the nominal level is a novel approach for overcoming the challenge that FDR involves the number of rejections - a random quantity. By up-weighting the smaller means when the rejection region is large and the larger means when the rejection region is small, this is a powerful procedure for maximizing the chance of rejection. The method can be particularly useful when prior information is present. Yet, we note that the power gained from the multi-weighting scheme may increase the FDR for two reasons: First, the step-up algorithm ignores the constraint (7) and FDR can be inflated for certain *W* and *m*. Especially in genomic studies, when *m* is large, this increases the chance that some corrected weights maybe much smaller than un-corrected ones. Ignoring the correction may cause FDR to rise above the nominal level. Second, in practice we cannot usually guess or estimate the non-centrality parameter *ξ*_*j*_ for false null hypotheses. Without relevant prior information, we can only use the data-splitting approach in Problem Setting I. This loss of sample size will also reduce the power. As suggest by Roeder and Wasserman [9], using a data-splitting approach and a weighted Bonferroni procedure may have less power than running un-weighted Bonferroni correction for the whole dataset. Therefore, we believe there is still room for improvement over the step-up procedure to address the above concerns.

### Problem setting III: grouped FDR control

Using the weighted wBH procedure and controlling FDR at level α, what is the *k* valued set of weights *W* = ( *W*_1_, *W*_2_⋯, *W*_*m*_) that will maximize the average power? Here, without the loss of generality, we assume $\;W_{1}=W_{2}=\cdots =W_{n_{1}}=w_{1}\text{,}\;W_{n_{1}+1}=W_{n_{1}+2\;}\cdots =W_{n_{1}+n_{2}}=w_{2},\cdots \text{,}\;W_{\sum_{j=1}^{j=k-1}n_{j}+1\;}=W_{\sum_{j=1}^{j=k-1}n_{j}+2\;}\cdots =W_{\sum_{j=1}^{j=k}n_{j}}=w_{k}\;\text{and}\;\sum_{j=1}^{j=k}n_{j}=m$.

This problem is motivated by Stratified False Discovery Rate (SFDR) control. Sun et al. [15] propose this method in the context of genetic studies when there is a natural stratification of the *m* hypotheses to be tested. For example, in a genetic study of the long-term complications of type I diabetes [16], researchers plan to screen about 1500 SNPs in candidate genes and identify SNPs that are associated with at least one out five phenotypes of interest. A total of 7500 tests will be carried out simultaneously, while natural stratification exists for these tests. Therefore, SFDR would be appropriate to account for this type of data.

SFDR controls FDR in each stratum. Let *α*_*j*_ denote the FDR in the *j*th stratum. To investigate the relationship between *α*_*j*_ and overall FDR *α*, based on the work of Storey [4], it can be shown that that $\;E\left(\frac{V}{R}\right)=E\left(V\right)/E\left(R\right)$ when tests are independent. Then

(8) $$\alpha =E\left(\frac{\sum_{j}V^{\left(j\right)}}{\sum_{j}R^{\left(j\right)}}\right)=\frac{\sum_{j}E\left(V^{\left(j\right)}\right)}{\sum_{j}E\left(R^{\left(j\right)}\right)}=\sum_{j}v_{j}\alpha^{\left(j\right)}\;$$

where *V*^(*j*)^ and *R*^(*j*)^ denote the number of false rejections and total rejections in *j*th stratum, and $\;v_{j}=E\left(R^{\left(j\right)}\right)/\sum_{l}E\left(R^{\left(l\right)}\right)\;$. Since ∑_*j*_*ν*_*j*_ = 1, it is easy to show that when FDR in each stratum is controlled at level*α*, the overall FDR is controlled at *α*. The SFDR procedure can be implemented in the software package R using the function *p.adjust*.

To demonstrate the merit of SFDR, Sun et al. [15] describe a simulated genome-wide association study with 105,000 SNPs, among which 5,000 SNPs are from candidate genes and 100,000 SNPs are included to systematically scan the genome for novel associations. The number of associated genes in each stratum is assumed to be 100 and the power to detect a single true association is assumed to be 0.7 with Type I error of 0.001. If the FDR threshold is set to be 0.1, then SFDR is expected to identify 111 true associations as compared to 88 via traditional FDR. This simulation indicates that SFDR can take advantage of an imbalanced distribution of true signal across stratums.

SFDR is a special case of problem setting III. SFDR controls the FDR in each stratum at level α, while the weighted FDR only requires that the overall FDR be controlled at level α. This implies that the optimal weights derived from problem setting III will have better power than SFDR because of the greater degrees of freedom.

Problem setting III is still an open problem. We have not found any literature directly addressing this problem. Given the indirect solution in problem setting II, the optimal weight for this setting is not hard to estimate. The major difference between setting II and setting III is that setting III reduces the variance among the weights. It is not surprising that maximum achievable power in setting III is less than setting II, but setting III has at least two advantages over setting II: first, the weight estimate in setting III is more robust. Estimating the non-centrality parameters for each test, reduces the dimension of the parameter space and leads to more robust estimates. Second, it is possible to use all samples to estimate the unknown parameters rather than using a data-splitting approach that causes the power loss due to smaller sample size.

## Conclusion and discussion

We summarized three p-value weighting schemes. The first focuses on control of FWER while the second and third focus on FDR control. The latter two differ in the method of assigning weights. All three methods seek to identify weights that maximize statistical power.

Problem setting I, involving FWER control, is more tractable analytically than the other two and has been studied more extensively. FWER can be easily expressed in a functional form and identifying the optimal weight is reduced to a maximization problem. Roeder et al. [17] demonstrated application of this method to a genome-wide association study, illustrating how to combine the prior information and the weighted Bonferroni approach. This idea should be of high relevance, as the advent of modern biology has made extensive information on gene location and biochemical pathways available in the public domain. Effective use of such information may hold the key to success of genome-wide studies.

Problem setting II, controlling FDR while seeking optimal weights, remains unresolved. FDR involves a random term in the dominator, making the optimization problem difficult. Yet, the setting is an important one: FDR is widely accepted in genomic studies as a method for controlling false discovery with greater power than the Bonferroni method. Roquain and van de Wiel’s [14] novel multi-weight approach under fixed rejection region reduces the problem to one similar to setting I, suggesting that some results from the weighted Bonferroni method might be adopted for multi-weight FDR control as well.

The drawback of the multi-weighting approach is that the conditions required to achieve maximum power are stringent and hard to achieve in real situations. Our third problem setting, therefore, attempts to bring more robustness into the weighting scheme by using the stratified FDR method of Sun et al. [2009]. This method has more power than traditional FDR when the distribution of the true signal across stratums is highly skewed. Restricting the weight into *k* values controls FDR in a similar fashion as SFDR but has more flexibility. We view it as a complement between the relative conservative SFDR and the highly dynamic multi-weight approach. It also brings in a way to incorporate prior knowledge on grouping, such as genes from the same biological pathway or SNPs that are located adjacent to one other on the chromosome. This problem setting is the least well-developed of the three, but results from the other two are generally applicable. Therefore, we expect that SFDR will be a major near-term focus of research in weighted FDR.

In conclusion, we recommend setting III as the generally preferred approach for weighted hypothesis testing in genome-wide association studies. While setting I may be easier to implement, it is likely to be too conservative. Setting II controls FDR, which is a more relaxed requirement than setting I. However, it is difficult to identify optimal weights for FDR control. Roquain’s method requires many regulatory conditions that are hard to achieve in real situations. Setting III can incorporate prior knowledge on grouping as well as stabilize the dynamic weighting scheme by offering the same weight within groups. Therefore, we see this as a highly promising approach.

Future work should address the issue of dependence among hypothesis tests. In the context of genome-wide association studies, there may be strong correlations among different SNPs that violate the independence assumption of the BH and Bonferoni procedures. There are at least two approaches to addressing this problem: (1) principle component based methods [18-20], which focus on identifying the effective number of tests using matrix decomposition, and (2) permutation test methods [21-23], which use efficient algorithms that fully account for the correlation structure among SNPs. Both of these approaches indicate that adjusting for positive dependence typically results in a gain in power. We expect that these approaches can be naturally extended to weighted hypothesis testing to improve the procedures reviewed here.
